# Supplementary material for: SARS-CoV-2 E and 3a Proteins Are Inducers of Pannexin Currents
Source: Cells. 2023 May 25;12(11):1474. doi: 10.3390/cells12111474 (PMC10252541; doi:10.3390/cells12111474)
Supplement: Supplementary file 1 [file cells-12-01474-s001.zip › cells-2310720-supplementary.pdf]

SARS-CoV-2 E and 3a proteins are inducers of pannexin currents

Barbara B. R. Oliveira-Mendes [1,†], Malak Alameh [1,2,†], Béatrice Ollivier [1], Jérôme Montnach [1], Nicolas Bidère [3,4], Frédérique Souazé [5], Nicolas Escriou [6], Flavien Charpentier [1], Isabelle Baró [1], Michel De Waard [1,2,*,‡] and Gildas Loussouarn [1,*,‡]

1 l'institut du thorax, Nantes Université, CNRS, INSERM, F-44000 Nantes, France;

2 Labex Ion Channels, Science and Therapeutics, F-06560 Valbonne, France

3 Team SOAP, CRCI2NA, INSERM, CNRS, Nantes Université, Université d'Angers, F-44000 Nantes, France

4 Equipe Labellisée Ligue Contre le Cancer, F-75006 Paris, France

5 CRCI2NA INSERM, CNRS, Nantes Université, F-44000 Nantes, France

6 Département de Santé Globale, Institut Pasteur, Université Paris Cité, F-75015 Paris, France

* Correspondence: michel.dewaard@univ-nantes.fr (M.D.W.); gildas.loussouarn@univ-nantes.fr (G.L.);

Tel.: +33-228-080-076 (M.D.W.)

† These authors contributed equally to this work.

‡ These authors contributed equally to this work.

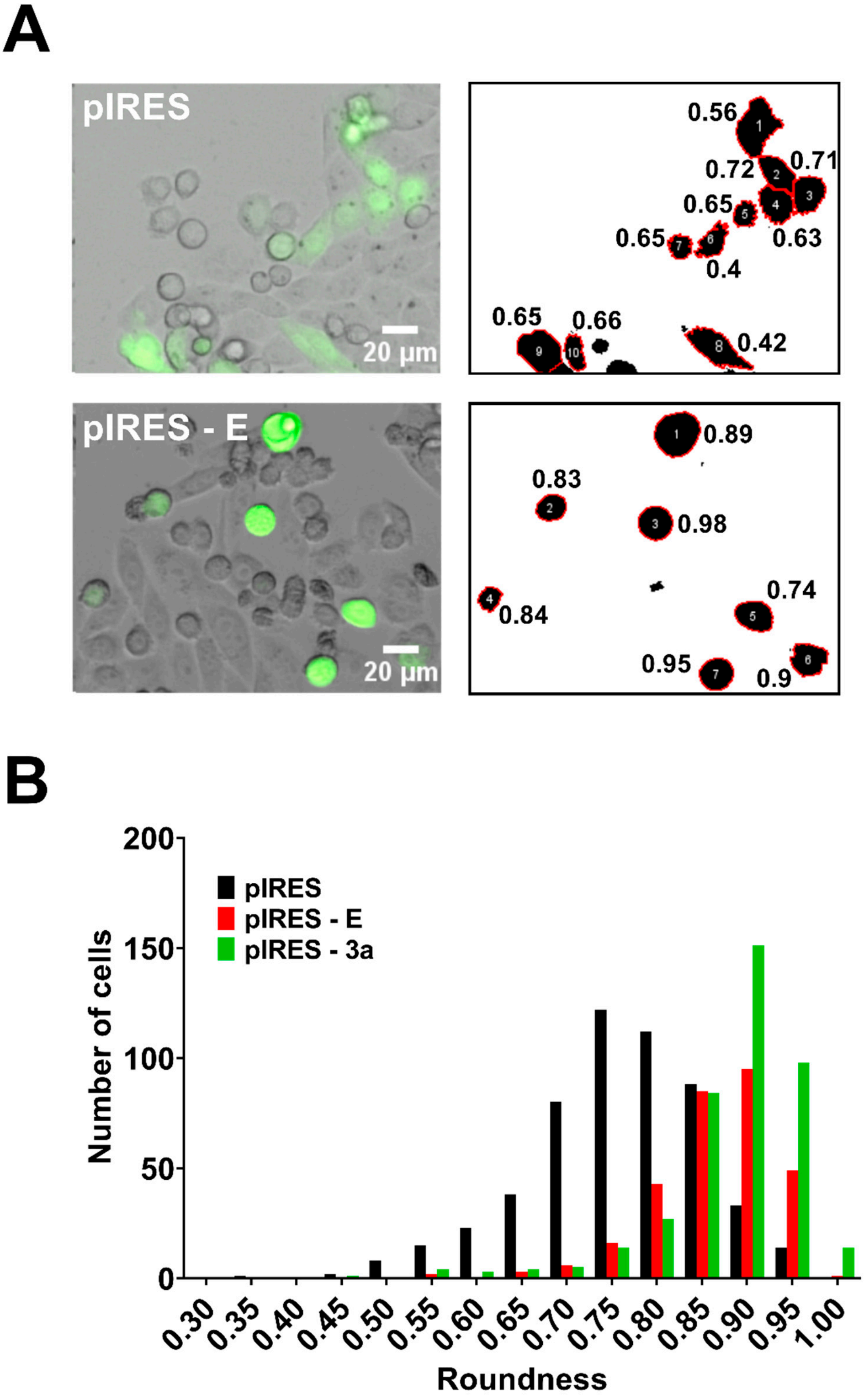

Supplementary Figure S1: Morphology analysis of cells transfected with control pIRES, pIRES - E or pIRES - 3a plasmids. **(A) Left:** example of superimposed brightfield and eGFP fluorescence images. **Right:** Automatic particle analysis showing the roundness index. **(B)** Distribution of cells roudnesses from the analysis of 5 areas of 1.7 mm x 1.7 mm for cells transfected with control pIRES, pIRES - E or pIRES- 3a plasmids.

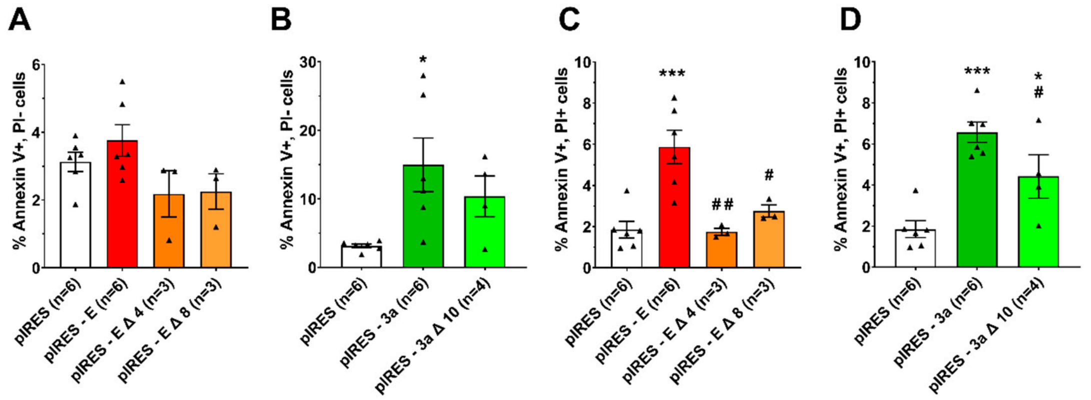

Supplementary Figure S2: Effects of E or 3a protein expression on early (Annexin V+, PI- in A&B) and late (Annexin V+, PI+ in C&D) cell death. Mean percentage (± sem) of stained cells among eGFP-positive CHO cells expressing only eGFP (pIRES), or eGFP and full-length or truncated E or 3a protein. * $p<0.05$, *** $p<0.001$ as compared to pIRES control, one-way ANOVA, ## $p<0.01$, # $p<0.05$ as compared to full-length protein, t-test.

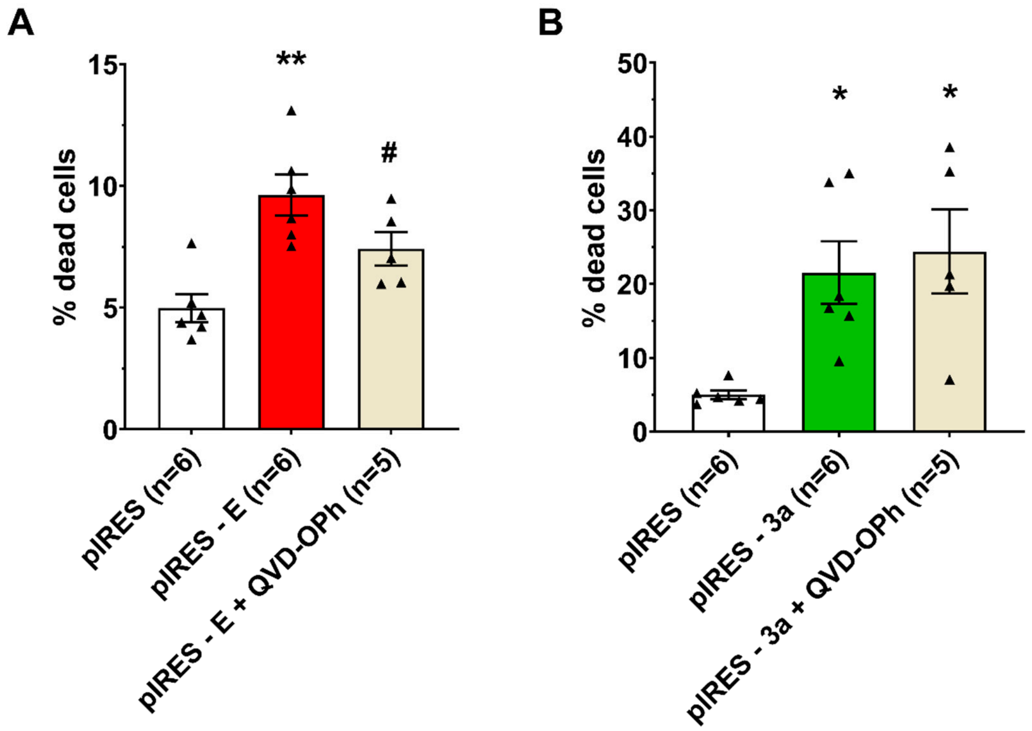

Supplementary Figure S3: Caspase dependence of E and 3a protein-induced cell death. (**A**) Cell death induced by E protein expression is reduced by the pan-caspase inhibitor QVD-OPh. Flow cytometry analysis of eGFP-positive CHO cells expressing only eGFP (pIRES), or co-expressing eGFP and the full-length E protein (pIRES - E), in absence or presence of the pan-caspase inhibitor QVD-OPh. ** $p<0.01$, as compared to pIRES control, one-way ANOVA. # $p<0.05$ as compared to E protein, t-test. (**B**) Cell death induced by 3a protein expression is not reduced by the pan-caspase inhibitor QVD-OPh. * $p<0.05$, as compared to pIRES control, one-way ANOVA. Concentrations are indicated in the method section.

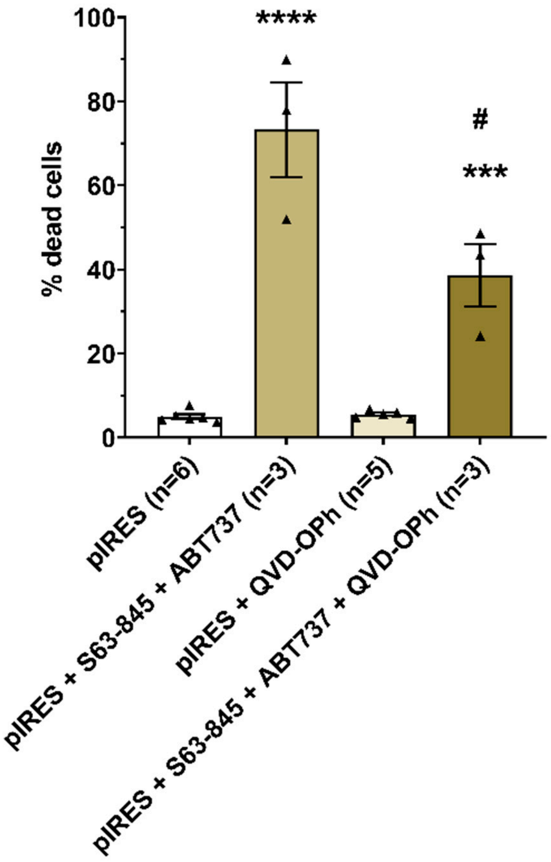

Supplementary Figure S4: Test of the apoptosis inducers and inhibitor in CHO cells. Flow cytometry analysis of eGFP-positive CHO cells expressing only eGFP (pIRES), in absence and or presence of the apoptosis inducers S63-845+ABT737 and inhibitor QVD-OPh. **** p<0.0001, *** p<0.001, as compared to pIRES control, one-way ANOVA, # p<0.05 as compared to pIRES+S63-845+ABT737, t-test.

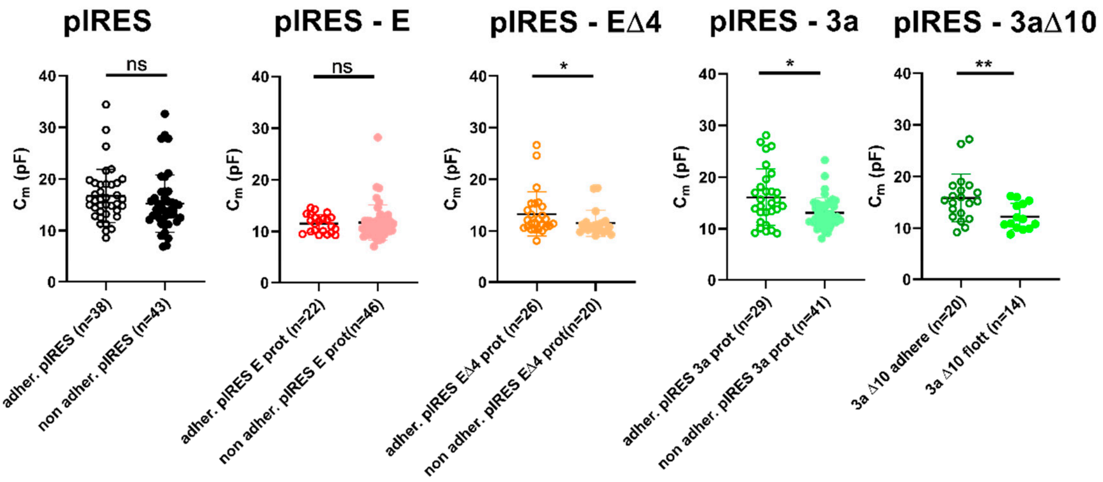

Supplementary Figure S5. Effect of expression of E or 3a protein on membrane capacitance in adhering *vs.* non-adhering CHO cells. * p<0.05, ** p<0.01, as compared to adhering cells, Mann Whitney test.

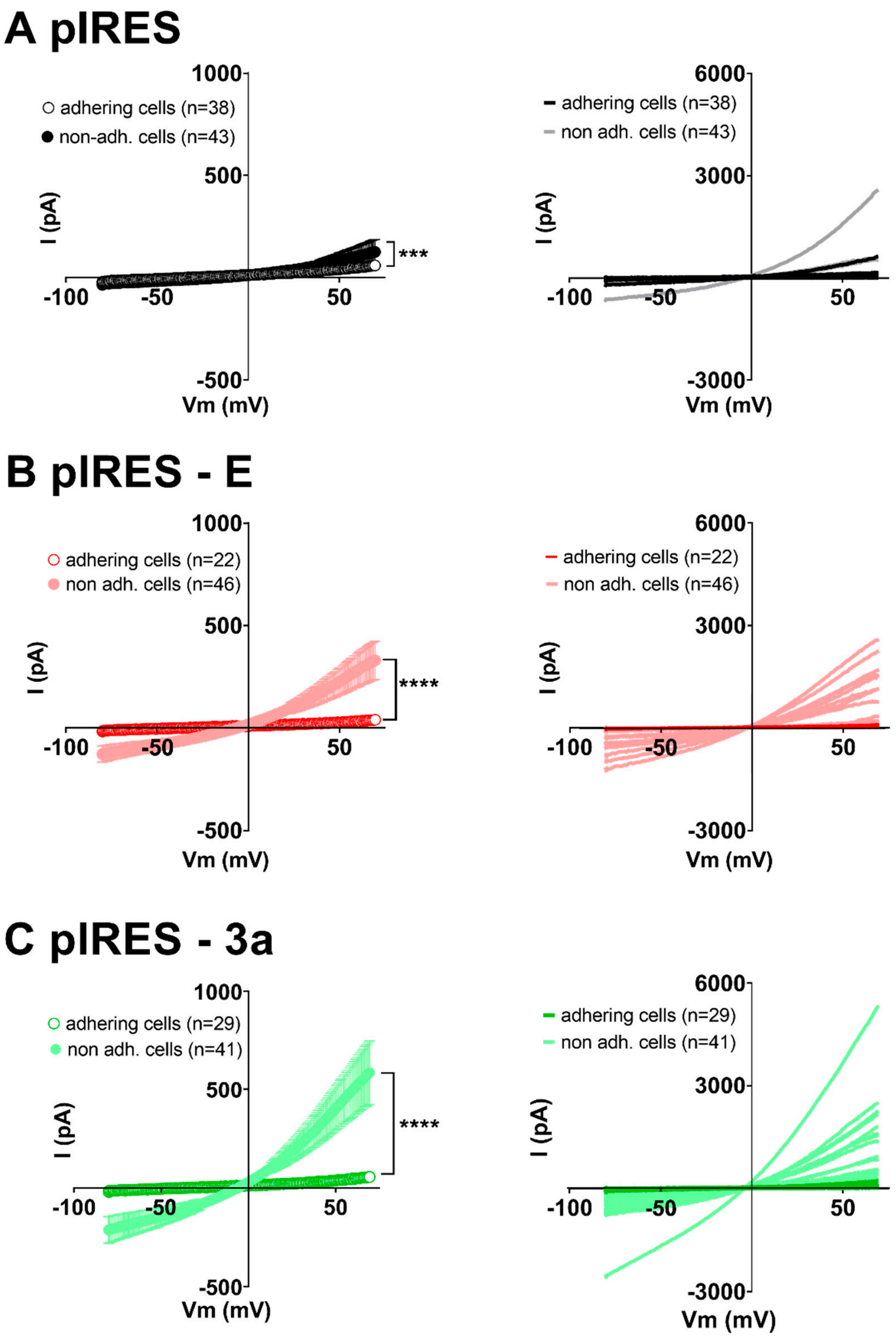

Supplementary Figure S6. Expression of E or 3a protein is accompanied by outwardly rectifying currents in non-adhering CHO cells only. Same as Figure 3, with current amplitudes shown instead of current densities. **** p<0.0001, *** p<0.001, as compared to adhering cells, two-way ANOVA on Ranks.

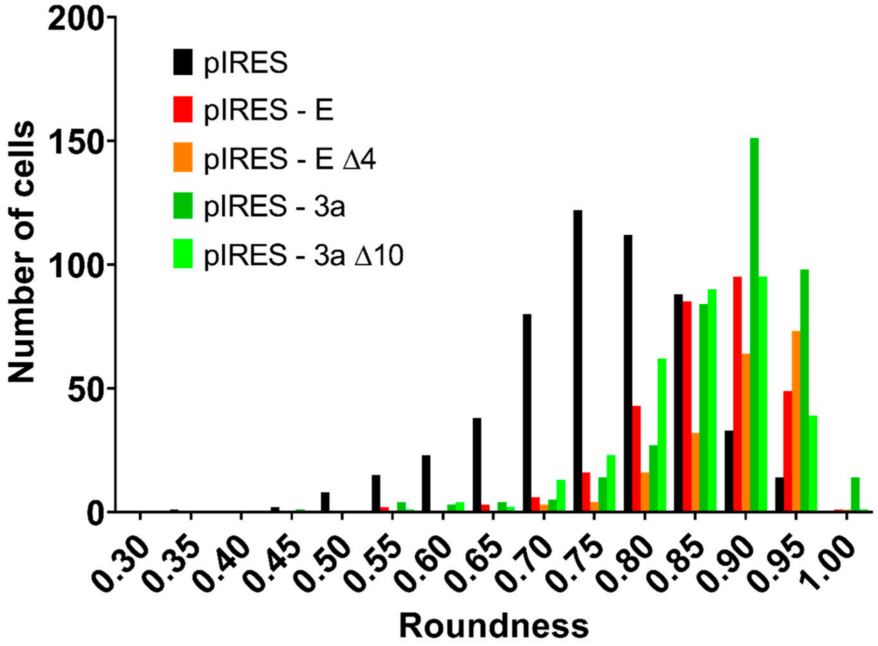

Supplementary Figure S7. Distribution of cells roundness. From the analysis of 5 areas of 1.7 mm x 1.7 mm of transfected CHO cells (Cf. Figure 6A).

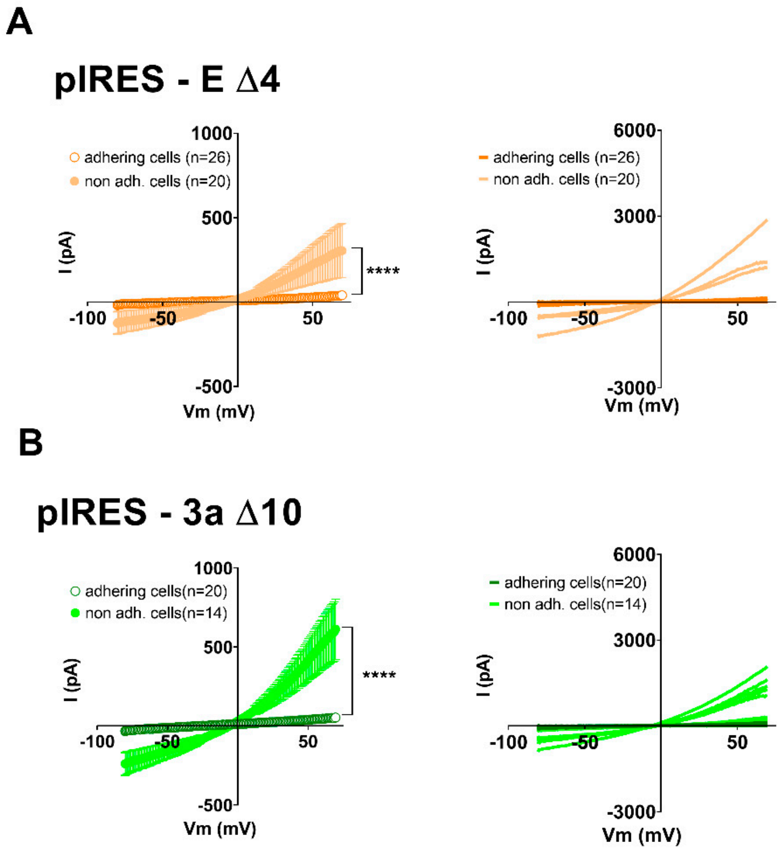

Supplementary Figure S8. C-terminal deletion of E or 3a protein does not prevent cell characteristic changes. Same as Figure 6B&C, with current amplitudes shown instead of current densities. **** p<0.0001, as compared to adhering cells, two-way ANOVA on Ranks.
